# Author Correction: Identification and characterization of GLDC as host susceptibility gene to severe influenza

**DOI:** 10.1038/s44321-024-00164-5

**Published:** 2025-07-09

**Authors:** Jie Zhou, Dong Wang, Bosco Ho-Yin Wong, Cun Li, Vincent Kwok-Man Poon, Lei Wen, Xiaoyu Zhao, Man Chun Chiu, Xiaojuan Liu, Ziwei Ye, Shuofeng Yuan, Kong-Hung Sze, Jasper Fuk-Woo Chan, Hin Chu, Kelvin Kai-Wang To, Kwok Yung Yuen

**Affiliations:** 1 State Key Laboratory of Emerging Infectious Diseases, The University of Hong Kong, Pokfulam, Hong Kong; 2 Department of Microbiology, The University of Hong Kong, Pokfulam, Hong Kong; 3 Research Centre of Infection and Immunology, The University of Hong Kong, Pokfulam, Hong Kong; 4 Carol Yu Centre for Infection, The University of Hong Kong, Pokfulam, Hong Kong; 5 The Collaborative Innovation Center for Diagnosis and Treatment of Infectious Diseases, The University of Hong Kong, Pokfulam, Hong Kong; 6 Department of Clinical Microbiology and Infection Control, The University of Hong Kong-Shenzhen Hospital, Shenzhen, China

## Abstract

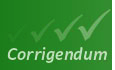

**Correction to:** *EMBO Molecular Medicine* (2018) 11:e9528. 10.15252/emmm.201809528 | Published online 28 November 2018

The authors contacted the journal upon discovering a subpanel was incorrectly selected in Figure 6E. After discussions with the authors and conducting its own analysis, the journal corrects the following figure panel.

**Figure 6E is withdrawn and replaced**.

**Source data for all Figure 6E panels is published with this correction**.

**Figure 6E:**
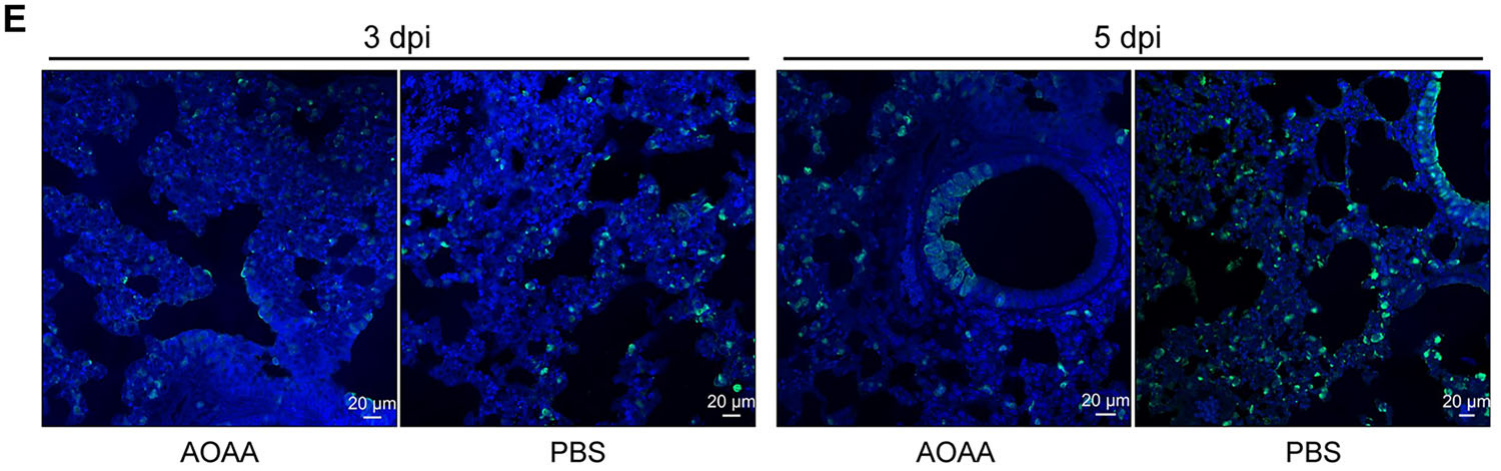
Original. Source data are available online for this figure.

**Figure 6E:**
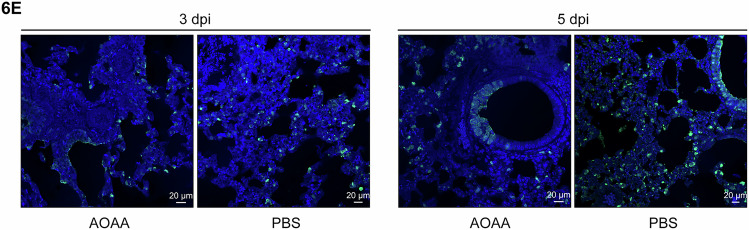
Corrected. Source data are available online for this figure.

Author statement:

The image labelled AOAA at 3 dpi (the first image from the left of Figure 6E) was wrongly selected when we prepared the figure for publication. The image was incorrectly taken from the lung tissue AOAA at 5 dpi image (third panel from the left of Figure 6E).

The original image of the lung tissue slide from an AOAA-treated mouse at 3 dpi is provided to correct the published image.

This error does not affect the interpretation of the data or the conclusions of the manuscript.

All authors agree to this author correction.

## Supplementary information

Corrected Figure 6E Source Data

Original Published Figure 6E Source Data

