# Supplementary material for: Identification and characterization of GLDC as host susceptibility gene to severe influenza
Source: EMBO Mol Med. 2018 Nov 28;11(1):e9528. doi: 10.15252/emmm.201809528 (PMC6328914; doi:10.15252/emmm.201809528)
Supplement: Supplementary file 1 — Appendix [file EMMM-11-e9528-s001.pdf]

Appendix PDF

Table of contents

1. Appendix Table S1.
2. Appendix Supplementary Methods

Appendix Table S1. Primer sequences for detection of mRNA expression of host genes.

Human primers		Mouse primers	
hIFI6-F	TCGCTGATGAGCTGGTCTGC	mIFNα-F	TGATGAGCTACTACTGGTCAGC
hIFI6-R	ATTACCTATGACGACGCTGC	mIFNα-R	GATCTCTTAGCACAAGGATGGC
hOAS1-F	CATCCGCCTAGTCAAGCACTG	mIFNβ-F	CAGCTCCAAGAAAGGACGAAC
hOAS1-R	CACCACCCAAGTTTCCTGTAG	mIFN-R	GGCAGTGTAACTCTTCTGCAT
hIFIT1-F	GCAGCCAAGTTTTACCGAAG	mIFITM3-F	CCCCCAAACTACGAAAGAATCA
hIFIT1-R	GCCTTTCTCCGAAGTTTCCT	mIFITM3-R	ACCATCTTCCGATCCCTAGAC
hIFIT3-F	AAAAGCCCAACAACCCAGAAT	mIFI35-F	AGCCAGCCAGATAACCACAG
hIFIT3-R	CGTATTGGTTATCAGGACTCAGC	mIFI35-R	CCGGGTCTCTACATCCCCA
hMX1-F	GCCAGGACCAGGTATACAG	mIFI27-F	GACTCTCCGTGCCATCTACTG
hMX1-R	GCTCCTTCAGGAGCCAGA	mIFI27-R	CCTCTATCGCCATATCTGCCAC
hIFITM3-F	TGTCCAAACCTTCTTCTCTCC	mOAS1-F	GTGAAGGATCTTAGCAGCACC
hIFITM3-R	CGTCGCCAACCATCTTCC	mOSA1-R	AAAGCACCTATGAAGTCCACG
hTRIM22-F	GGATCGTCAGTAGAGATGCTGC	mIFIT1-F	ATCGCGTAGACAAAGCTCTTC
hTRIM22-R	GAACTTGCAGCATCCCACTCAG	mIFIT1-R	GTTTCGGGATGTCCTCAGTTG
hISG15-F	GAGAGGCAGCGAACTCATCT	mISG15-F	GGTGTCCGTGACTAACTCCAT
hISG15-R	AGGGACACCTGGAATTCGTT	mISG15-R	CTGTACCACTAGCATCACTGTG
hPKR-F	TCTTCATGTATGTGACACTGC	mMX1-F	GACCATAGGGGTCTTGACCAA
hPKR-R	CACACAGTCAAGGTCCTTAG	mMX1-R	AGACTTGCTCTTTCTGAAAAGCC
hIFI27-F	TGC CTC GGG CAG CCT		
hIFI27-R	TTG GTC AAT CCG GAG AGT CC		
hIFI35-F	AACAAAAGGAGCACACGATCA		
hIFI35-R	CTCCGTTCCTAGTCTTGCCAA		
hIFI6-F	TCGCTGATGAGCTGGTCTGC		
hIFI6-R	ATTACCTATGACGACGCTGC		
hIFNβF	CAACTTGCTTGGATTCCTACAAAG		
hIFNβR	TATTCAAGCCTCCCATTCAATTG		
hIFNα-F	CTTGAAGGACAGACATGACTTTGGA		
hIFNα-R	GGATGGTTTCAGCCTTTTGGA		
hGAPDH-F	GGAGCGAGATCCCTCCAAAAT		
hGAPDH-R	GGCTGTTGTCATACTTCTCATGG		

Appendix Supplementary Methods

Detection of thymidine with LC-MS/MS.

A549 cells were seeded in 6-well plate with a confluence of 10^6 cells/well. After overnight culture, increasing doses of GLDC inhibitor AOAA was supplemented in the culture medium and maintained for 48 hours. Subsequently, the cells were collected, accurately counted and washed with ice-cold PBS and lyophilized. All the extraction steps were performed on ice as described previously (Buchel, Rhyn et al., 2013). The cells were resuspended with 2.5ml of 10:1 ethyl acetate-2-propanol (v/v), vortexed on a rotary mixer for 15min at 45rpm, followed by centrifugation at 3000g for 10min. The supernatant was transferred and dried in clean 1.5ml polypropylene Eppendorf tube. Subsequently, 2ml of organic phase was added to the original sample to repeat the extraction. The dried sample was reconstituted using 200mL of 0.1% formic acid and sonicated for 30s. The extract was centrifuged at 2000g for 5min and the clear supernatant was transferred to HPLC autosampler vials for analysis. In each group, at least triplicate samples were analyzed.

LC-MS/MS analysis was performed using M-class UHPLC (Waters, USA) and a Synapt G2-Si HDMS spectrometer (Waters, USA) with multiple reaction monitoring (MRM) mode. An aliquot of 8μl of sample was analyzed. LC separation was performed on an Agilent Eclipse Plus C18 column (2.1×100 mm, 1.8μm). The mobile phase A contains 1.0mM ammonium acetate and 0.1% formic acid in water, while solvent B consists of 100% acetonitrile. A flow rate of 0.2ml/min at 45ºC was applied. The gradient program ran as follows: 0.5min (0.5% solvent B), 4 min (99% solvent B), 9 min (99% solvent B). The column was re-equilibrated by holding 0.5% solvent B between 9 to 10 min before the next sample injection. The ion source was operating in positive mode with a capillary voltage at 2000V and a source temperature of 120ºC. The desolvation temperature was set at 350ºC. Nitrogen gas was used for instrument operation and the desolvation gas was set at 800L/Hr. The sample cone and source offset voltage were set at 80V and 120V, respectively. Thymidine quantification was achieved by measuring the peak intensity of Q1/Q3=243.2/127.2 transition. Data were processed with Masslynx software (V4.1, Waters, USA).

Evaluation of AOAA toxicity in vitro and in vivo.

The possible toxicity of AOAA was assessed in A549 cells. A549 cells were incubated with indicated concentration of AOAA at 37°C for 48 hours. The cell-free culture medium was applied to MTT assay as described elsewhere (Zhou, Chu et al., 2014). After anesthesia via intraperitoneal injection of ketamine/xylazine cocktail containing 70~100mg/kg ketamine and 10~20mg/kg xylazine, 2 groups of 3 female Balb/c mice of 6~8 weeks old were in were intranasally administered with AOAA (10 mg/kg weight) or PBS in a volume of 20μl twice per day. Mouse body weight was daily monitored for 5 days.

References

Buchel B, Rhyn P, Schurch S, Buhr C, Amstutz U, Largiader CR (2013) LC-MS/MS method for simultaneous analysis of uracil, 5,6-dihydrouracil, 5-fluorouracil and 5-fluoro-5,6-dihydrouracil in human plasma for therapeutic drug monitoring and toxicity prediction in cancer patients. *Biomed Chromatogr* 27: 7-16

Zhou J, Chu H, Li C, Wong BH, Cheng ZS, Poon VK, Sun T, Lau CC, Wong KK, Chan JY, Chan JF, To KK, Chan KH, Zheng BJ, Yuen KY (2014) Active replication of Middle East respiratory syndrome coronavirus and aberrant induction of inflammatory cytokines and chemokines in human macrophages: implications for pathogenesis. *J Infect Dis* 209: 1331-42
