# Supplementary material for: Identification and characterization of GLDC as host susceptibility gene to severe influenza
Source: EMBO Mol Med. 2018 Nov 28;11(1):e9528. doi: 10.15252/emmm.201809528 (PMC6328914; doi:10.15252/emmm.201809528)
Supplement: Supplementary file 2 — Expanded View Figures PDF [file EMMM-11-e9528-s002.pdf]

# Expanded View Figures

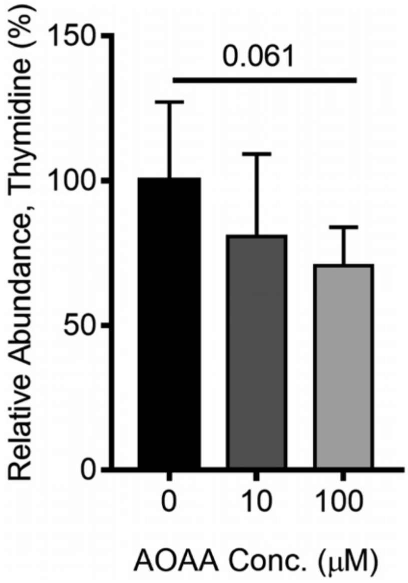


**Figure EV1. Dose-dependent inhibition of cellular thymidine in the AOAA-treated A549 cells.**

A549 cells were treated with the indicated concentrations of AOAA. After 48 h, cells were harvested for the detection of the relative amount of thymidine with LC-MS/MS. Data shown are representative of two independent experiments, $n = 5$. Graphs show mean ± SD. Unpaired $t$-test is used for data analysis.

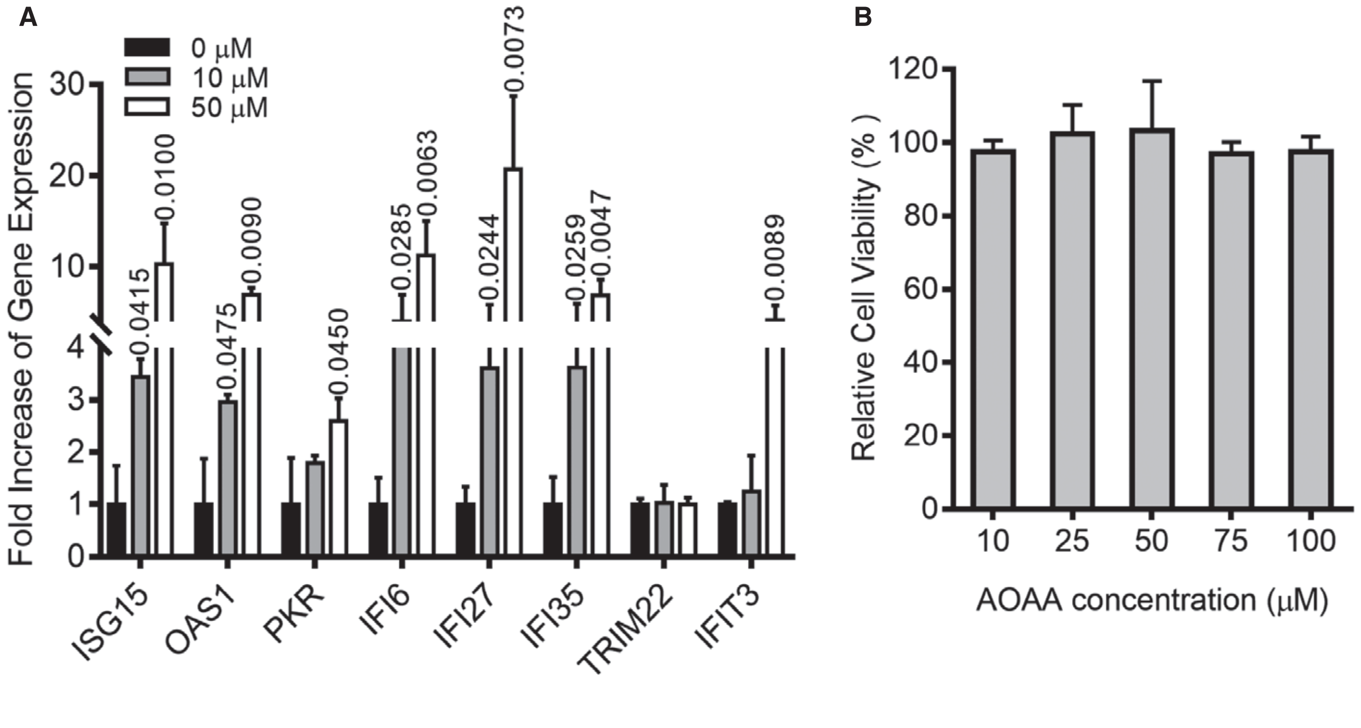


**Figure EV2. AOAA induced the heightened ISGs at 48 h after H7N9 infection and cell viability assay of AOAA.**

A A549 cells treated with AOAA (0, 10, 50 μM) were then inoculated H7N9 virus before and after a MOI of 0.01 inoculation. At 48 hpi, the cells were harvested for detecting expression levels of ISGs. Data shown are representative of three experiments, $n = 3$. Graphs show mean ± SD. Unpaired $t$-test is used for data analysis.

B The effect of AOAA on cell viability was assessed in A549 cells. A549 cells were incubated with the indicated concentration of AOAA at 37°C for 48 h. The cell-free culture medium was applied to MTT assay. Data shown are representative of two experiments, $n = 3$. Graphs show mean ± SD. Unpaired $t$-test is used for data analysis.

© 2018 The Authors

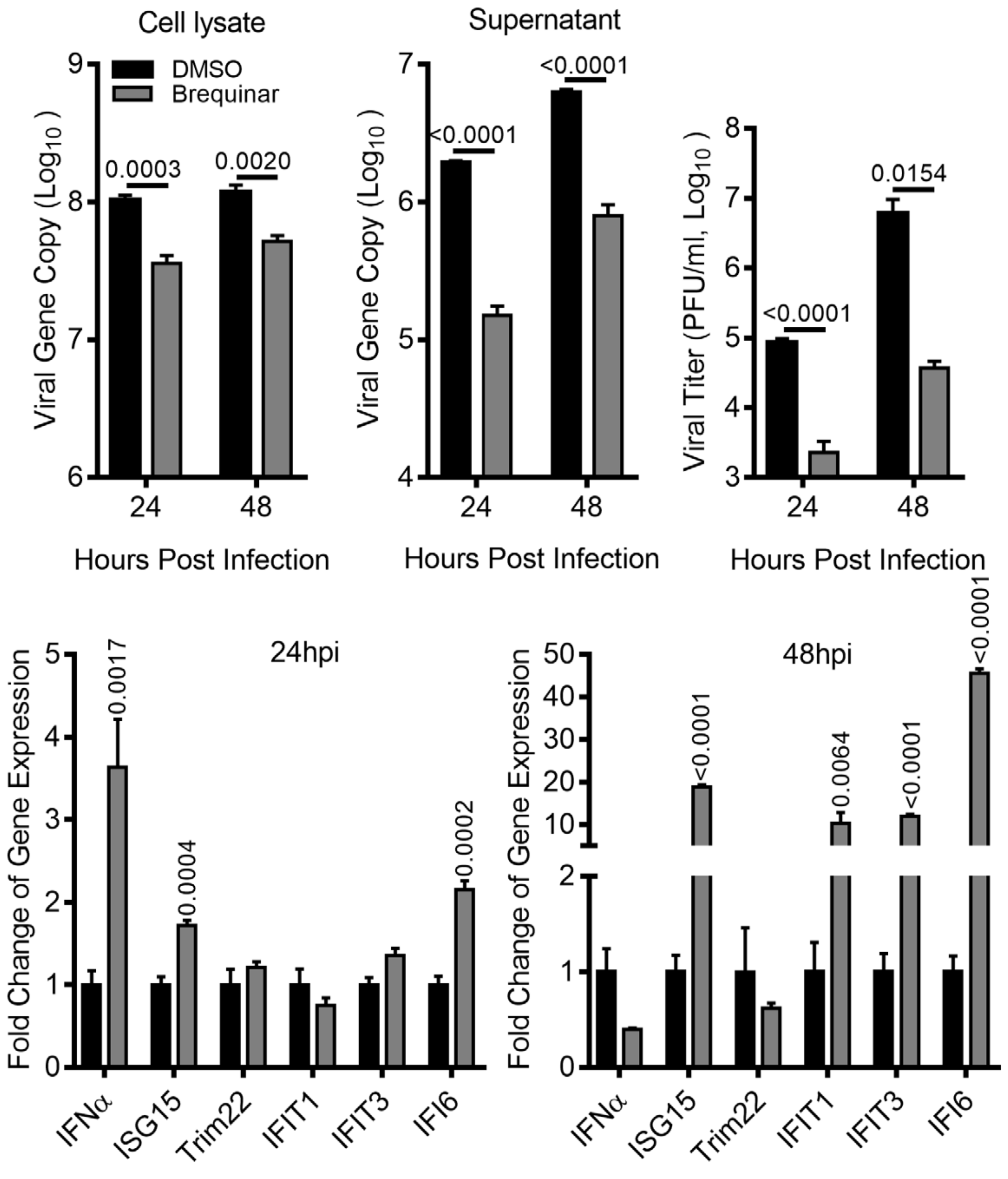


**Figure EV3. The effect of brequinar on antiviral immunity and viral replication.**

After 1-h pretreatment of 10 μM brequinar (Tocris Biosciences, Cat No. 6196) or DMSO, A549 cells were inoculated with H7N9 virus with a MOI of 0.01 then were maintained in DMEM with 2 μg/ml TPCK-trypsin, 3% BSA and 10 μM brequinar or DMSO. At the indicated time points, cell lysates and cell-free media were collected for detection of viral load, cellular gene expression, and viral titration. Data shown are representative of two experiments, $n = 3$. Graphs show mean ± SD. Unpaired $t$-test is used for data analysis.


© 2018 The Authors

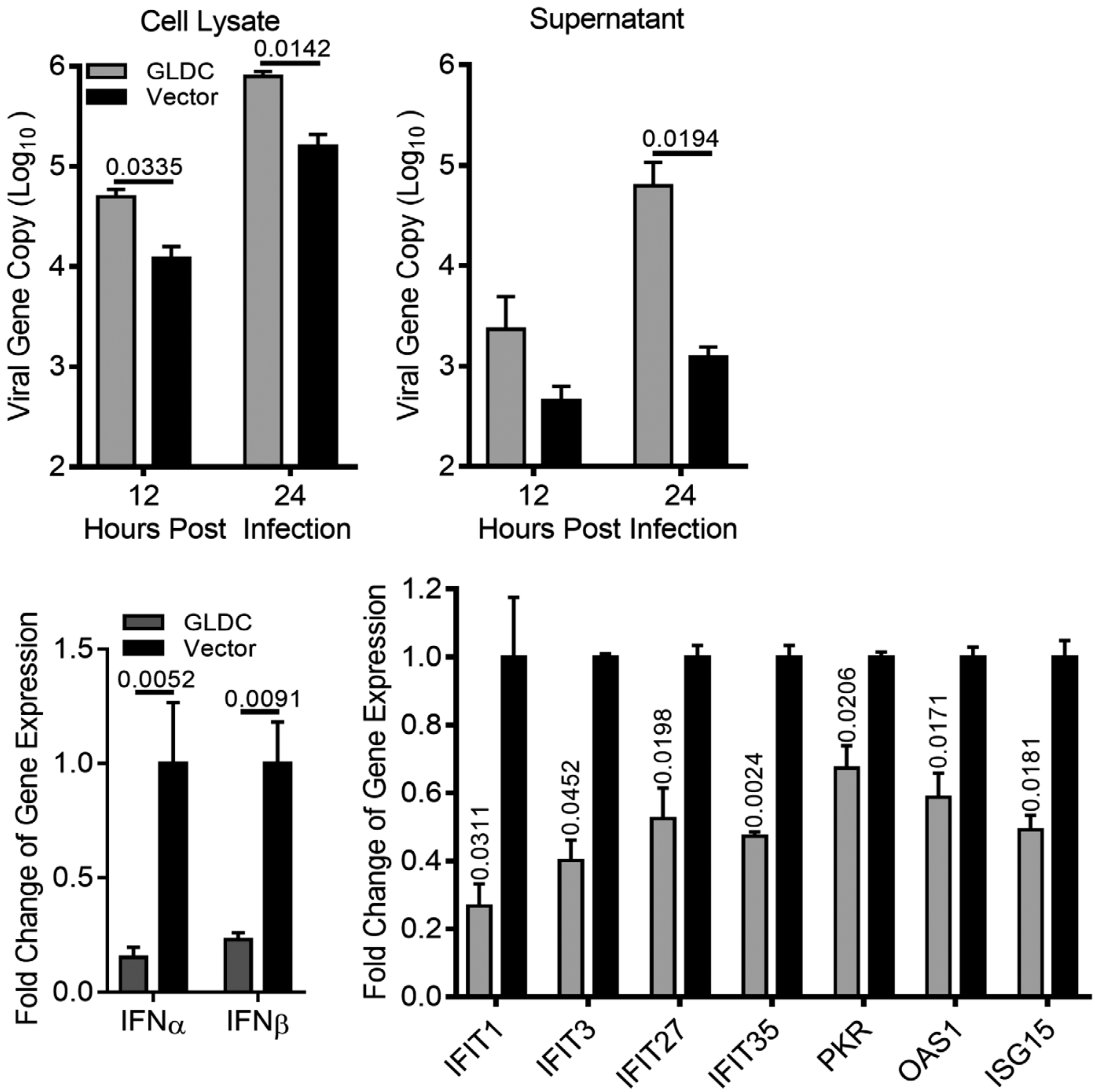


**Figure EV4. GLDC overexpression promoted replication of H1N1 via the attenuated antiviral response.**

A549 cells transfected with pcDNA3.1-GLDC or pcDNA3.1-His A vector were inoculated with H1N1 virus at an MOI of 0.01. At the indicated hpi, cell lysates and cell-free supernatants were harvested for viral load detection. The cell lysates collected at 12 hpi were used to measure the expression levels of antiviral genes. Data shown are representative of three experiments, $n$ = 3. Graphs show mean ± SD. Unpaired $t$-test is used for data analysis.

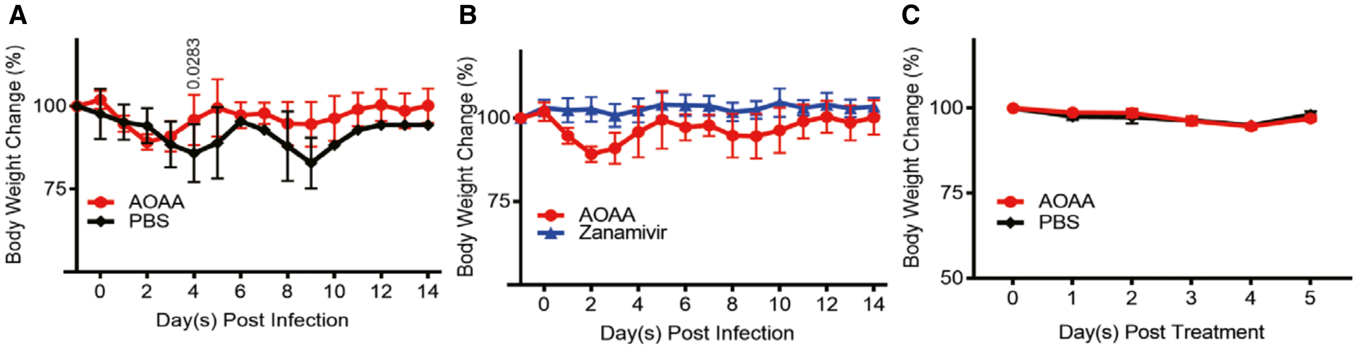


**Figure EV5. Mouse body weight change during infection and evaluation of AOAA toxicity in vivo.**

A, B The body weight change of AOAA-, zanamivir-, and PBS-treated mice ($n = 9$) after virus inoculation.

C Body weight change of the mice intranasally administered with AOAA ($n = 3$, 10 mg/kg body weight) and PBS ($n = 3$).

Data information: Unpaired *t*-test is used for data analysis. Graphs show mean ± SD.

 © 2018 The Authors
