# Supplementary material for: Identification and characterization of GLDC as host susceptibility gene to severe influenza
Source: EMBO Mol Med. 2018 Nov 28;11(1):e9528. doi: 10.15252/emmm.201809528 (PMC6328914; doi:10.15252/emmm.201809528)

The source images in Figure 5E

Confirmation of GDLC depletion after siRNA depletion.

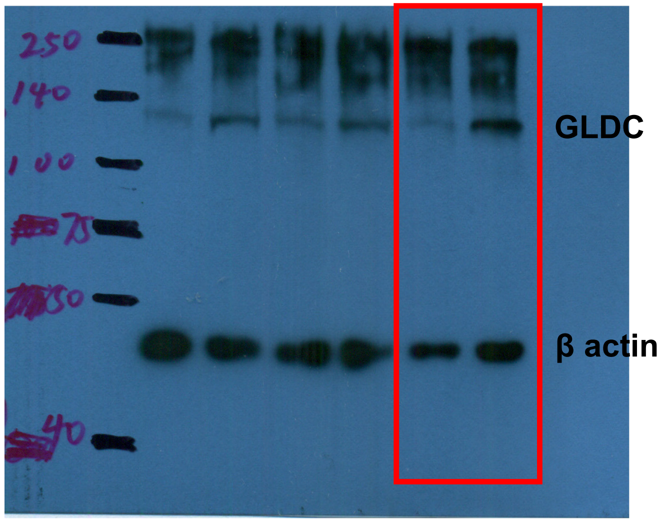

Confirmation of GDLC overexpression at 24 and 48 hours post infection.

Flipped scan (presented in paper)

The original scan

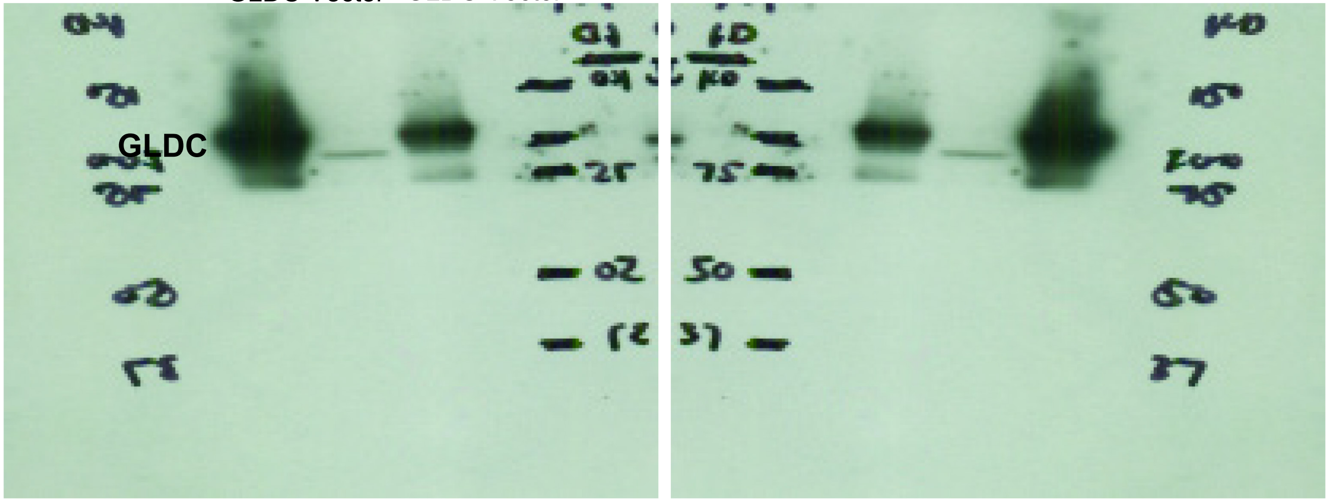

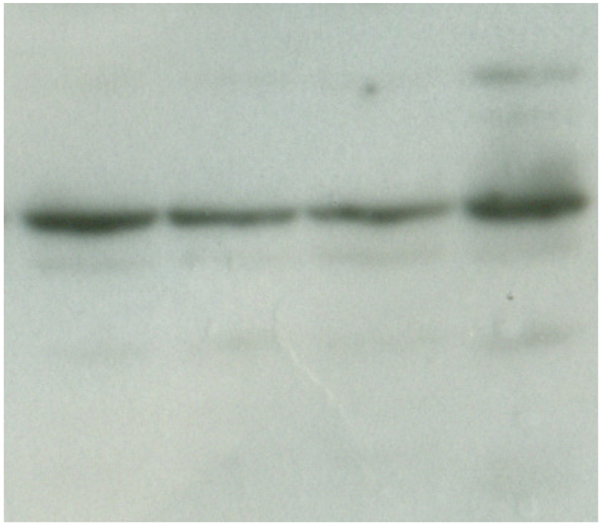

Supplement: Supplementary file 4 — Source Data for Figure 5E [file EMMM-11-e9528-s003.pdf]
