# Supplementary figures and images for: Author Correction: Identification and characterization of GLDC as host susceptibility gene to severe influenza
Source: EMBO Mol Med. 2025 Jul 9;17(8):2163–4. doi: 10.1038/s44321-024-00164-5 (PMC12340071; doi:10.1038/s44321-024-00164-5)

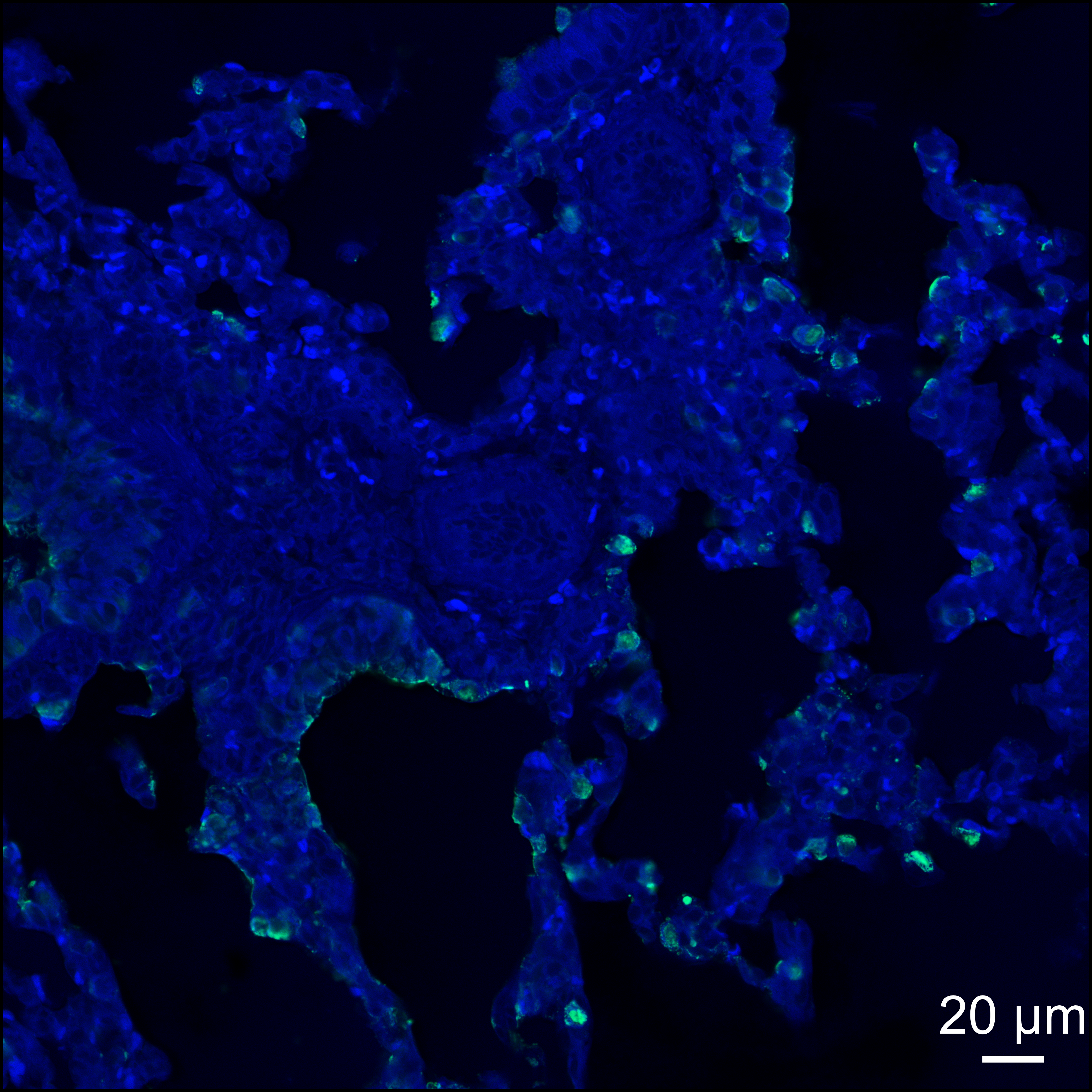

Supplement: Supplementary file 1 — Corrected Figure 6E Source Data [file 44321_2024_164_MOESM1_ESM.zip › AOAA-3dpi.tif]

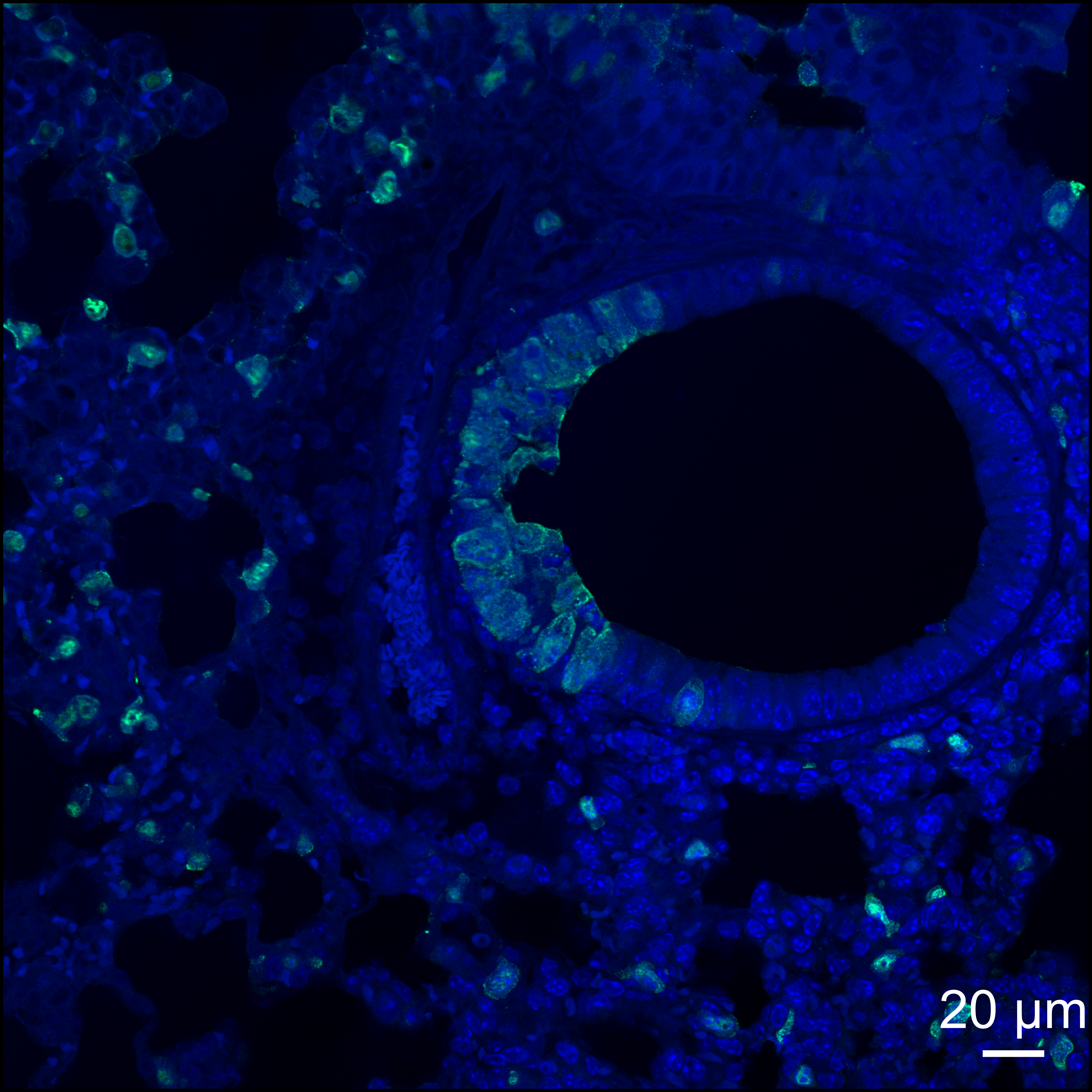

Supplement: Supplementary file 1 — Corrected Figure 6E Source Data [file 44321_2024_164_MOESM1_ESM.zip › AOAA-5dpi.tif]

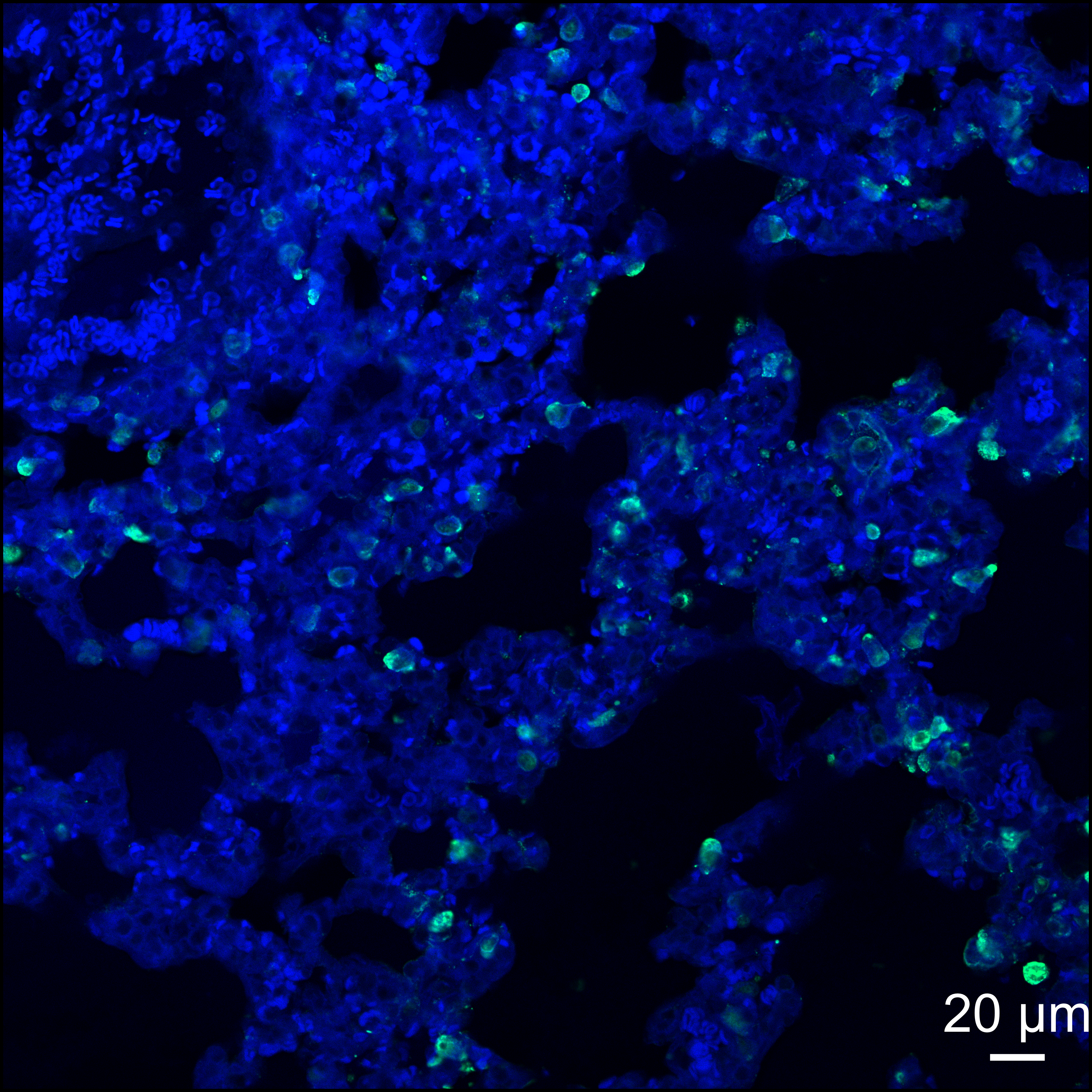

Supplement: Supplementary file 1 — Corrected Figure 6E Source Data [file 44321_2024_164_MOESM1_ESM.zip › PBS-3dpi.tif]

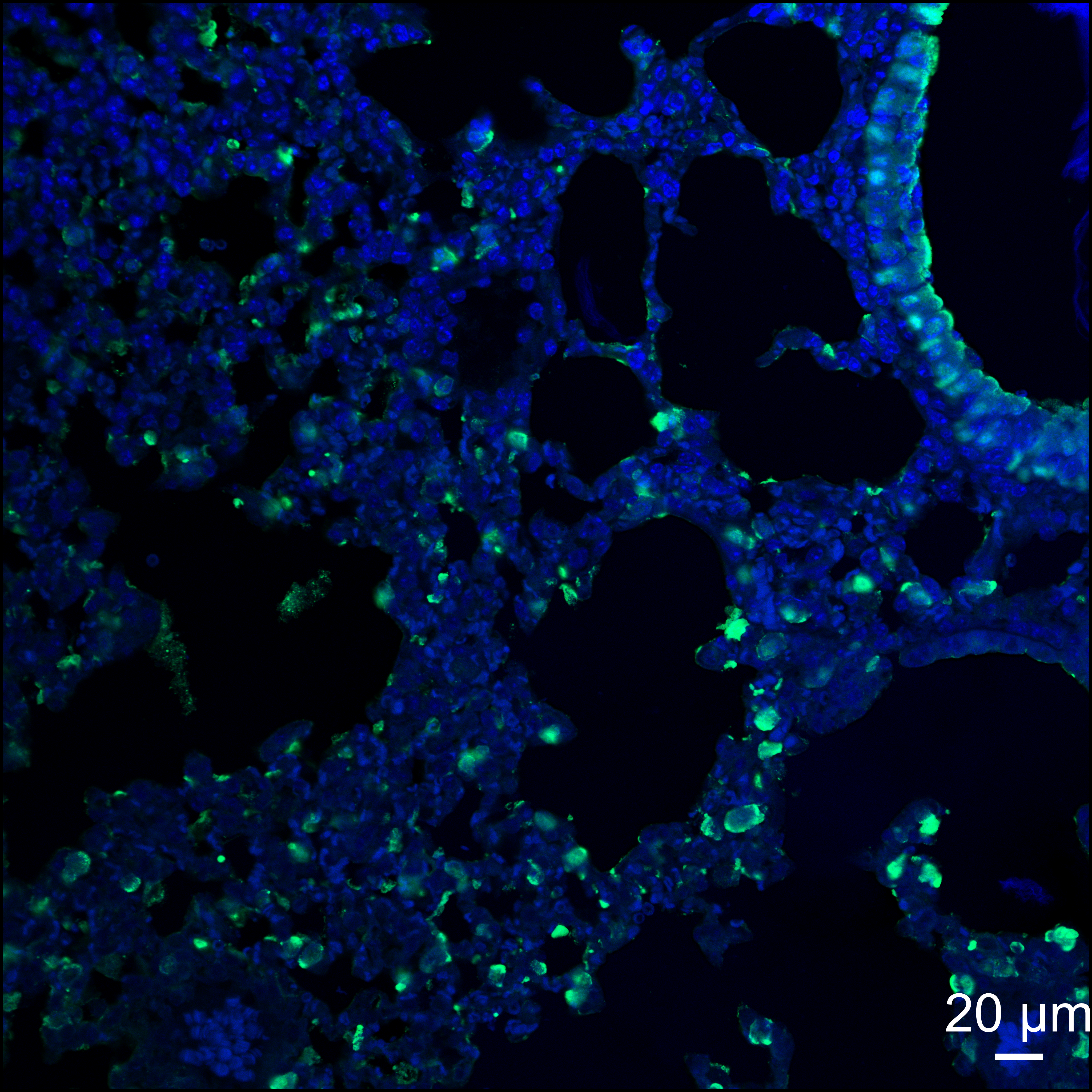

Supplement: Supplementary file 1 — Corrected Figure 6E Source Data [file 44321_2024_164_MOESM1_ESM.zip › PBS-5dpi.tif]

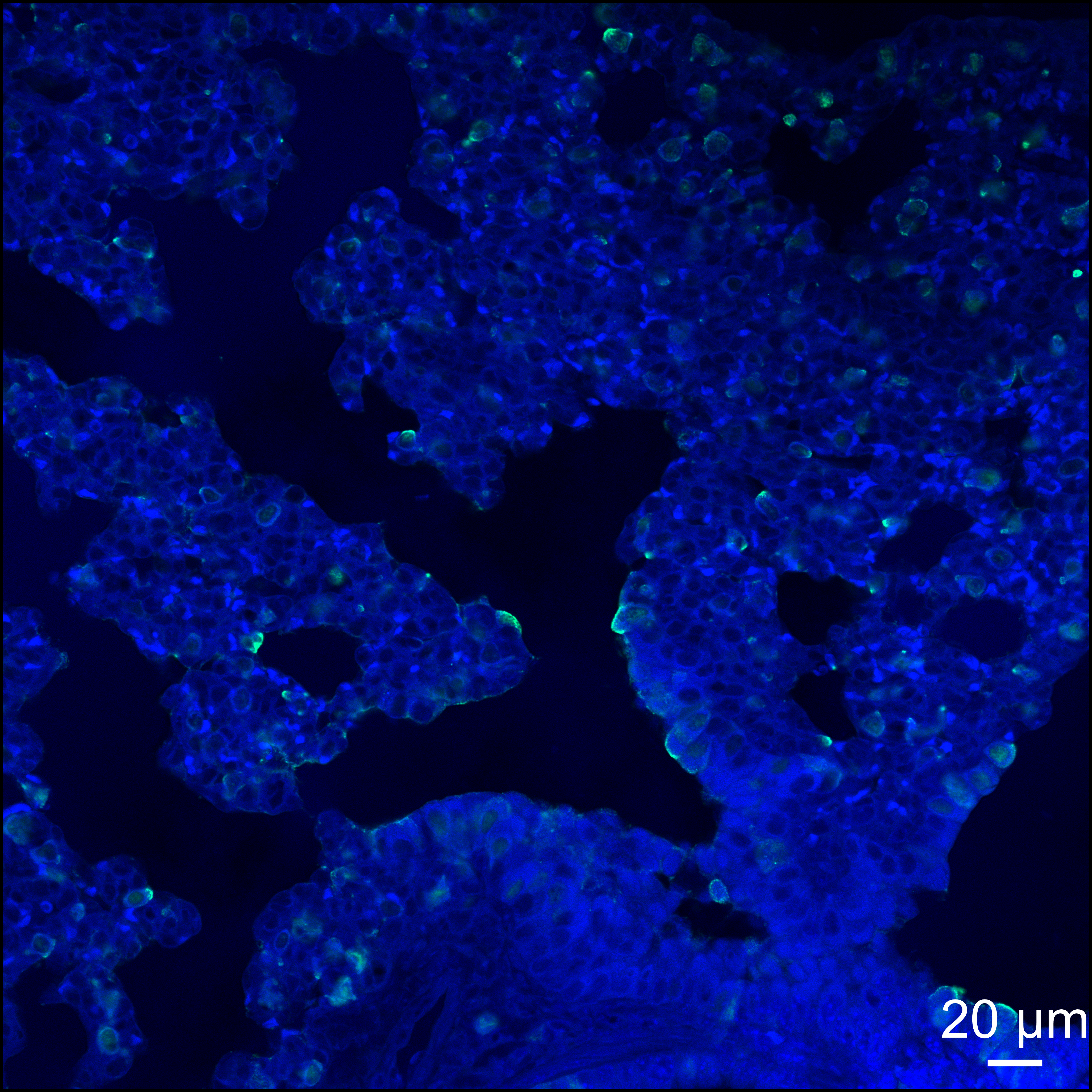

Supplement: Supplementary file 2 — Original Published Figure 6E Source Data [file 44321_2024_164_MOESM2_ESM.zip › AOAA-3dpi.tif]
